# A comparison between triplet and doublet chemotherapy in improving the survival of patients with advanced gastric cancer: a systematic review and meta-analysis

**DOI:** 10.1186/s12885-019-6294-9

**Published:** 2019-11-20

**Authors:** Xinjian Guo, Fuxing Zhao, Xinfu Ma, Guoshuang Shen, Dengfeng Ren, Fangchao Zheng, Feng Du, Ziyi Wang, Raees Ahmad, Xinyue Yuan, Junhui Zhao, Jiuda Zhao

**Affiliations:** 1 Affiliated Hospital of Qinghai University, Affiliated Cancer Hospital of Qinghai University, Xining, 810000 China; 2 Shouguang Hospital of Traditional Chinese Medicine, Weifang, 262700 China; 3 Department of Medical Oncology, Cancer hospital, Chinese academy of medical sciences, Peking Union Medical College, Beijing, 100021 China; 4 Peking University Cancer Hospital and Institute, Beijing, 100142 China

**Keywords:** Advanced gastric cancer, Triplet chemotherapy, Doublet chemotherapy, Meta-analysis, First-line chemotherapy

## Abstract

**Background:**

Chemotherapy can improve the survival of patients with advanced gastric cancer. However, whether triplet chemotherapy can further improve the survival of patients with advanced gastric cancer compared with doublet chemotherapy remains controversial. This study reviewed and updated all published and eligible randomized controlled trials (RCTs) to compare the efficacy, prognosis, and toxicity of triplet chemotherapy with doublet chemotherapy in patients with advanced gastric cancer.

**Methods:**

RCTs on first-line chemotherapy in advanced gastric cancer on PubMed, Embase, and the Cochrane Register of Controlled Trials and all abstracts from the annual meetings of the European Society for Medical Oncology (ESMO) and the American Society of Clinical Oncology conferences up to October 2018 were searched. The primary outcome was overall survival, while the secondary outcomes were progression-free survival (PFS), time to progress (TTP), objective response rate (ORR), and toxicity.

**Results:**

Our analysis included 23 RCTs involving 4540 patients and 8 types of triplet and doublet chemotherapy regimens, and systematic review and meta-analysis revealed that triplet chemotherapy was superior compared with doublet chemotherapy in terms of improving median OS (HR = 0.92; 95% CI, 0.86–0.98; *P* = 0.02) and PFS (HR = 0.82; 95% CI, 0.69–0.97; *P* = 0.02) and TTP (HR = 0.92; 95% CI, 0.86–0.98; *P* = 0.02) and ORR (OR = 1.21; 95% CI, 1.12–1.31; *P* < 0.0001) among overall populations. Compared with doublet chemotherapy, subgroup analysis indicated that OS improved with fluoropyrimidine-based (HR = 0.80; 95% CI, 0.66–0.96; *P* = 0.02), platinum-based (HR = 0.75; 95% CI, 0.57–0.99; *P* = 0.04), and other drug-based triplet (HR = 0.79; 95% CI, 0.69–0.90; *P* = 0.0006) chemotherapies while not with anthracycline-based (HR = 0.70; 95% CI, 0.42–1.15; *P* = 0.16), mitomycin-based (HR = 0.81; 95% CI, 0.47–1.39; *P* = 0.44), taxane-based (HR = 0.91; 95% CI, 0.81–1.01; *P* = 0.07), and irinotecan-based triplet (HR = 1.01; 95% CI, 0.82–1.24; *P* = 0.94) chemotherapies. For different patients, compared with doublet chemotherapy, triplet chemotherapy improved OS (HR = 0.89; 95% CI, 0.81–0.99; *P* = 0.03) among Western patients but did not improve (HR = 0.96; 95% CI, 0.86–1.07; *P* = 0.47) that among Asian patients.

**Conclusions:**

Compared with doublet chemotherapy, triplet chemotherapy improved OS, PFS, TTP, and ORR in patients with advanced gastric cancer in the population overall, and improved OS in Western but not in Asian patients.

## Background

Gastric cancer is a significant health burden worldwide. Global Cancer Statistics 2018 estimates that there will be 1,033,701 (5.7% of all sites) new cases and 782,685 (8.2 of all sites) deaths due to gastric cancer in 2018 [1]. Generally, 80–90% of patients with gastric cancer are diagnosed at an advanced stage, implying that the tumor either cannot be resected through operation or developed a recurrence or metastasis after surgery [2, 3]. The prognosis of these patients remains very poor, and the median survival time is only about 12 months [3]. Several targeted therapies, such as the human epidermal growth factor receptor 2 (HER2) antibody trastuzumab and the anti-vascular endothelial growth factor receptor 2 drugs including ramucirumab and apatinib, and immunotherapies including pembrolizumab and nivolumab have shown efficacy in metastatic gastric cancer [4, 5]. Though molecularly targeted treatment is promising for improving the survival of patients with advanced gastric cancer, the number of patients who appropriately receive this treatment is less considering the high heterogeneity and lack of targets in gastric cancer. Therefore, systemic chemotherapy remains the current main treatment in patients with advanced gastric cancer [6]. Especially for first-line setting, only trastuzumab or ramucirumab combined with chemotherapy is approved, with only about 10% of patients experiencing HER2 overexpression [7].

Chemotherapy can improve the survival of patients with advanced gastric cancer. Compared with best supportive care, systemic chemotherapy improves not only the survival but also the quality of life of the patients [2, 8]. According to the number of chemotherapeutic drugs included in the treatment method, chemotherapy regimens of patients with advanced gastric cancer are usually divided into singlet, doublet, and triplet chemotherapy. Combination chemotherapy has substantially higher objective response and survival rates than monotherapy [2, 8]. However, whether triplet chemotherapy can improve the survival of patients with advanced gastric cancer compared with doublet chemotherapy remains controversial considering the discrepancy among studies [2, 4, 8]. To date, nearly 30 studies have focused on this issue. Meta-analyses also show inconsistent results. For instance, one meta-analysis concludes that taxane-based triplet chemotherapy improves the survival of patients with advanced gastric cancer than doublet chemotherapy, while another meta-analysis does not support this [8, 9].

Several major international guidelines for advanced gastric cancer also have different recommendations concerning triplet or doublet chemotherapy. The European Society for Medical Oncology (ESMO) guidelines of 2016 state that both doublet and triplet chemotherapies belong to level I and grade A corresponding to levels of evidence and grades of recommendation, respectively, in patients with advanced gastric cancer [10]. However, the National Comprehensive Cancer Network guidelines (version 2.2018) suggest that doublet regimens are preferred and triplet regimens should be reserved for medically fit patients with good performance status (PS) [4]. Additionally, the Japanese gastric cancer treatment guidelines 2014 (version 4) only classifies triplet regimen as category 3, implying that cannot be used in general practice [5]. The Chinese Society of Clinical Oncology guidelines for the diagnosis and treatment of primary gastric cancer (2018 edition) also suggest that triplet chemotherapy is an “optional strategy” but not a “basic strategy” [11]. With all of these uncertainties regarding the role of triplet regimen, as evidenced by the different guidelines discussed above, there is an urgent appeal of a new study on the definite role of triplet regimen in advanced gastric cancer. Such studies are still ongoing and have been published [12–14]. Nevertheless, two recent large-scale studies convey contrasting results. Wang et al. reported that modified DCF (docetaxel and cisplatin plus fluorouracil) regimen improved progression-free survival (PFS) and overall survival (OS) in patients with treatment-naive advanced gastric cancer compared with cisplatin plus fluorouracil regimen [14]. Yasuhide Yamada et al. concluded that another modified DCF regimen (docetaxel and cisplatin plus S1) did not improve the OS of patients with untreated advanced gastric cancer compared with cisplatin plus S1 regimen [12].

Hence, whether triplet or doublet chemotherapy improves the survival of patients with advanced gastric cancer is still questionable in a first-line setting. Therefore, we conducted a systematic review and updated the meta-analysis of all published eligible randomized controlled trials (RCTs) to compare the efficacy, prognosis, and toxicity of triplet with doublet chemotherapy in patients with advanced gastric cancer.

## Methods

### Study protocol

The protocol of this systematic review has been registered on PROSPERO in September 2018 (registration, CRD42018110550).

### Literature search

We searched PubMed, Embase, and the Cochrane Register of Controlled Trials (CENTRAL) up to October 2018. Studies were selected using the following search terms: “gastric or esophagogastric or gastroesophageal or gastroesophagus or stomach,” “cancer or neoplasm or carcinoma or malignancy,” “chemotherapy or chemotherapeutic or antineoplastic agent or antineoplastic drug,” “randomized or randomised trial or randomized, controlled trial,” and free text searches. No language limits were applied. Results were limited to RCTs that compared OS, PFS, objective response rate (ORR), and safety between triplet and doublet chemotherapy in patients with advanced gastric cancer. Additionally, all abstracts from the annual meetings of the ESMO and the American Society of Clinical Oncology (ASCO) conferences up to October 2018 were also searched. The eligible reports were independently identified by two reviewers (XFM and FXZ), and disagreements were discussed with a third reviewer (DFR) until consensus was reached. This systematic review was conducted according to the Preferred Reporting Items for Systematic Reviews and Meta-Analyses (PRISMA) statement [15–17].

### Study selection

Studies meeting the following criteria of eligibility were included: 1) studies utilizing prospective phase II or III RCTs; 2) studies whose patients have pathologically proven advanced, recurrent, metastatic, or unresectable adenocarcinoma of the stomach or gastroesophageal junction; 3) studies with first-line chemotherapy setting; and 4) studies that compared at least two arms that consisted of the following chemotherapeutic drugs: fluoropyrimidine (F, either 5-fluorouracil [5-FU], capecitabine [Cap], or S-1), platinum (cisplatin [Cis] and oxaliplatin [Ox]), taxane ([T] and paclitaxel), anthracycline (doxorubicin [D] and epirubicin [E]), irinotecan (I), etoposide (E), semustine (Me), mitomycin (MMC), methotrexate (Mtx), uracil (U), or tegafur (Te). Studies that are retrospective or included patients receiving targeted treatment were excluded.

### Data extraction and quality assessment

The primary outcome was OS, defined as the time from the date of random assignment to the date of death or last date of follow-up. Secondary outcomes were PFS; time to progress (TTP), defined as the duration from the date of random assignment to the date of events occurring; ORR, which estimates the rate of complete response plus partial response; and grade 3 to 4 adverse events (AEs). Treatment-related AEs defined the highest grade of toxicity per patient. AEs data, when available, were recorded if scored as grade 3–4 toxicity.

The methodological quality of all eligible studies was assessed using the Cochrane Risk of Bias Tool (version 5.1.0) [18, 19].

### Statistical analysis

Survival analyses were conducted using the intention-to-treat (ITT) population. A fixed effects model was used to calculate the pooled hazard ratio (HR) estimate. HRs for progression and death were combined using an inverse-variance method based on a logarithmic conversion; 95% confidence intervals (95% CIs) were used to determine the standard error (SE), using the following formula: SE = 95% CI/1.96. Statistical heterogeneity was tested with the Cochran Q test and quantified by the *I*^2^ index. Heterogeneity was considered statistically significant when *P* is less than 0.05 or *I*^2^ is greater than 50%. A random effects model was carried among trials with significant heterogeneity; otherwise, a fixed effects model was used. Publication bias was tested using funnel plots. When comparing triplet versus doublet chemotherapy, subgroup analyses including whether the regimens included fluorouracil (FU), platinum, anthracycline, taxane, irinotecan (I), MMC, and others and whether the studies included either Asian or Western patients were prespecified in advance in the registered protocol. Furthermore, the subgroup analysis comparing different chemotherapy combinations only included those triplet regimens having two generic drugs available in doublet regimens and investigated the effectiveness of irinotecan-based chemotherapy regimen in improving the survival of patients with gastric cancer considering the rarity of irinotecan-based study. RevMan v5.3 software was used to report all outcomes. All tests were performed two-sided, with a *P* value less than 0.05 considered statistically significant.

## Results

### Literature search and study characteristics

**Fig. 1:**
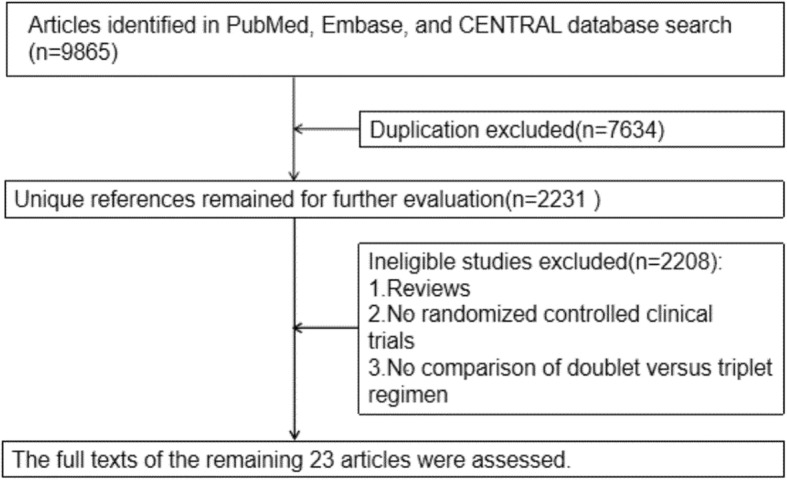
A flowchart of study selection

A total of 9865 unique references were identified through searching PubMed, Embase, and the CENTRAL. After the exclusion of duplicate publications, 2231 unique references remained for further evaluation. Of these papers, 2207 were excluded because of the following reasons: these papers were solely reviews, RCTs were not available for these papers, and these papers did not compare doublet versus triplet regimen. The full texts of the remaining 24 articles were assessed. Ultimately, 23 articles involving 4540 patients with advanced gastric cancer were included in our systematic review [12, 14, 20–40]. A flowchart of study selection is shown in Fig. 1.

Table 1 shows the characteristics of the studies included in this meta-analysis. Generally, 23 studies were included. The total number of included patients in every study ranged from 25 to 741. All RCTs satisfied the inclusion criteria and compared triplet combination versus doublet combination chemotherapy. Of the 23 included trials, two contained three groups, two triplet groups and one doublet group [24, 25]; one contained three groups, one triplet group and two doublet groups [27]; one contained four groups, two triplet groups and two doublet groups [29]; and the other were all two groups, one triplet group and one doublet group [12, 14, 20–23, 26, 28, 30–40].

**Table 1:** Characteristics of the subjects in eligible studies

Study	Number Arms	Efficacy				Age		Sex		Disease status				ECOG			
		OS	PFS	TTP	ORR	Median	Range	Male		LA		ME		0-1		≥2	
		Median months						N	%	N	%	N	%	N	%	N	%
Fluoropyrimidine-based																	
Ajani 2005	79 DTX+Cis+5-FU	9.6	5.9	NA	43	57	21-83	53	70	4	6	72	95	79	100	0	1
	76 DTX+Cis	10.5	5	NA	26	57	30-76	61	77	1	1	75	95	75	99	0	1
Douglass 1984	46 5-FU+Doxo+MMC	29.5	NA	NA	39	61.0	32-81	35	76	0	0	46	100	30	65	16	35
	46 Doxo+MMC	19	NA	NA	29	58	33-78	37	80	0	0	46	100	28	61	18	39
Roth 2007	41 DTX+Cis+5-FU	10.4	NA	4.6	36.6	61	35-78	30	73	2	5	39	95	41	100	0	0
	38 DTX+Cis	11.0	NA	3.6	18.4	58	40-70	29	76	7	18	31	82	38	100	0	0
Van Cutsem 2015	89 DTX+Ox+5-FU	14.6	7.6	NA	46.6	58	NA	61	69	0	0	89	100	87	98	2	2
	79 DTX+Ox	8.93	4.5	NA	23.1	59	NA	51	65	0	0	79	100	77	99	1	1
Platinum-based																	
Kikuchi K 1990	32 ADM+5-FU+Cis	NA	NA	NA	6	NA	NA	NA	NA	NA	NA	NA	NA	NA	NA	NA	NA
	33 ADM+5-FU	NA	NA	NA	0	NA	NA	NA	NA	NA	NA	NA	NA	NA	NA	NA	N
Park 2008	45 Cis+Iri+5-FU	10.5	6.2	NA	42	52	29-70	30	76	0	0	45	100	38	84	7	16
	46 Iri+5-FU	10.7	4.8	NA	42	55	26-73	30	67	0	0	45	100	35	78	11	29
Roth 1999	61 Epi+Cis+5-FU	9.6	NA	NA	42.6	54	NA	37	61	12	22	42	78	24	39	30	61
	61 Epi+5-FU	7.1	NA	NA	28.6	56	NA	42	69	16	30	40	84	27	44	29	56
Anthracyclin-based																	
Douglass 1984	39 5-FU+Doxo+Me	5.5	NA	NA	29	59.5	43-76	28	71	0	0	39	100	30	77	9	23
	48 5-FU+Me	3.3	NA	NA	14	62.0	24-79	38	80	0	0	48	100	35	72	13	28
Kim 2001	48 Epi+Cis+5-FU	8.5	NA	4.4	41.5	55	NA	45	75	3	5	57	95	54	90	6	10
	48 Cis+5-FU	7.3	NA	3.9	37.7	56	NA	42	70	3	5	57	95	53	88	7	12
KRGCGC 1992	25 Epi+Cis+5-FU	6.9	NA	NA	27	55	NA	45	75	3	5	57	97	54	90	6	10
	22 Cis+5-FU	4	NA	NA	24	55	NA	45	75	3	5	57	95	54	90	6	10
Yun 2010	44 Epi+Cis+Cap	NA	6.5	NA	37	55	37-51	28	64	NA	NA	NA	NA	40	91	1	9
	47 Cis+Cap	NA	6.4	NA	38	58	33-75	34	72	NA	NA	NA	NA	41	87	4	13
MMC-based																	
Cullinan 1985	51 5-FU+Doxo+MMC	NA	NA	NA	38.5	60	NA	39	76	20	39	31	61	32	63	19	37
	49 Doxo+5-FU	NA	NA	NA	27.7	63	NA	37	76	18	37	31	63	33	67	16	33
Koizumi 2004	33 5-DFUR+Cis+MMC	8.03	NA	NA	25	58	36-79	19	58	NA	NA	NA	NA	16	48	13	39
	29 5-DFUR+Cis	5.97	NA	NA	17.2	58	37-79	17	59	NA	NA	NA	NA	25	86	6	24
Taxane-based																	
AI-Batran 2013	79 DTX+Ox+5-FU	17.3	9.1	NA	48.6	69	65-81	51	71	22	31	50	69	67	93	5	7
	76 Ox+5-FU	14.5	7.1	NA	28.17	70	65-82	45	63	22	32	49	68	65	92	6	9
Van Cutsem 2006	227 DTX+Cis+5-FU	9.2	NA	5.6	37	55	26-79	159	72	6	3	213	96	218	99	3	1
	230 Cis+5-FU	8.6	NA	3.7	25	55	25-76	158	71	6	3	217	97	211	99	3	1
Wang 2015	119 DTX+Cis+5-FU	10.2	7.2	NA	48.7	56.6	19-80	81	68.1	30	25.2	89	77.4	115	96.6	4	3.4
	115 Cis+5-FU	8.5	4.9	NA	33.9	55.5	33-74	88	76.5	26	22.6	89	74.8	108	93.9	7	6.1
Yamada 2018	370 S-1+Cis	15.3	6.5	NA	56	NA	NA	NA	NA	NA	NA	NA	NA	NA	NA	NA	NA
	371 S-1+Cis+DOC	14.2	7.4	NA	59.3	NA	NA	NA	NA	NA	NA	NA	NA	NA	NA	NA	NA
Irinotecan-based																	
Guimbaud 2014	209 Epi+Cis+Cape	9.5	5.3	NA	39.2	61.4	28-84	154	74	36	17	173	83	169	81	36	17
	207 5-FU+Iri	9.7	5.8	NA	37.8	61.4	29-80	155	75	31	15	176	85	173	84	27	13
Lin 2009	13 5-FU+Ox+PTX	NA	NA	NA	62.5	55	36-67	18	72	NA	NA	NA	NA	NA	NA	NA	NA
	12 5-FU+Iri	NA	NA	NA	33.3	55	36-67	18	72	NA	NA	NA	NA	NA	NA	NA	NA
Other																	
Kim 1993	110 5-FU+Doxo+MMC	6.84	3	NA	25	54	19-77	68	62	NA	NA	NA	NA	75	68	23	21
	112 Cis+5-FU	8.61	5.5	NA	51	51	20-68	71	63	NA	NA	NA	NA	83	74	20	18
Li 2011	50 PTX+Cis+5-FU	10.8	NA	NA	48	59	20-74	32	68	22	46	28	56	NA	NA	NA	NA
	44 Ox+5-FU	9.9	NA	NA	45.5	58	20-75	31	70	17	41	27	61	NA	NA	NA	NA
Maiello 2011	36 Epi+Cis+Cap	NA	NA	NA	54.3	58	39-74	22	60	NA	NA	NA	NA	NA	NA	NA	NA
	31 DTX+5-FU	NA	NA	NA	22.6	61	44-75	23	74	NA	NA	NA	NA	NA	NA	NA	NA
Roth 2007	40 Epi+Cis+5-FU	8.3	NA	4.9	25	59	32-71	30	75	7	17	33	83	40	100	0	0
	41 DTX+Cis+5-FU	10.4	NA	4.9	36.6	61	35-78	30	73	2	5	39	95	41	10	0	0
Thuss-Patience 2005	45 Epi+Cis+5-FU	9.7	NA	5.5	35.6	63	33-75	36	80	1	2	44	98	44	98	1	2
	45 DTX+5-FU	9.5	NA	5.3	37.8	62	34-75	29	64	1	2	44	98	42	95	2	4
Van Hoefer 2000	133 5-FU+Doxo+MTX	6.7	3.3	NA	12	58	30-74	96	72	22	17	111	83	117	88	16	12
	134 Cis+5-FU	7.2	4.1	NA	20	57	24-74	91	68	21	16	113	84	114	85	20	15
	132 Eto+5-FU+LV	7.2	3.3	NA	9	59	25-74	90	68	22	17	110	83	120	92	12	9

*OS* Overall survival, PFS Progression-free survival, *TTP* Time to progression, *ORR* Objective response rate, *LA* Locally advanced, *ME* Metastatic disease, *ECOGE* Eastern Cooperative Oncology Group performance status, *NA* Not applicable, *DTX* Docetaxel, *DOC* Docetaxel, *PTX* Palictaxel,Ciscisplatin, *5- FU* Fluorouracil, *Cape* Capcapecitabine, *Cap* Capcapecitabine, *5-DFUR* Doxifluridine, *Ox* Oxaliplatin, *Doxo* Doxorubicin, *Epi* Epirubicin, *Iri* Irinotecan, *MMC* Mitomycin C, *Eto* Etoposide, *Cis* Cisplatin, *ADM* Adriamycin, *Me* Methyl-CCNU, *S-1* Tegafur

Of these studies, 2380 were assigned to the triplet and 2160 to the doublet group. Median age was 51 to 70 years. In these studies, 2039 and 2501 (44.9 and 55.1%, respectively) patients were Asians and Westerners, respectively. PS was well balanced in all studies. All patients had an ECOG PS of 0 or 1.

### Overall survival, progression-free survival, time to progress, and objective response rate

**Fig. 2:**
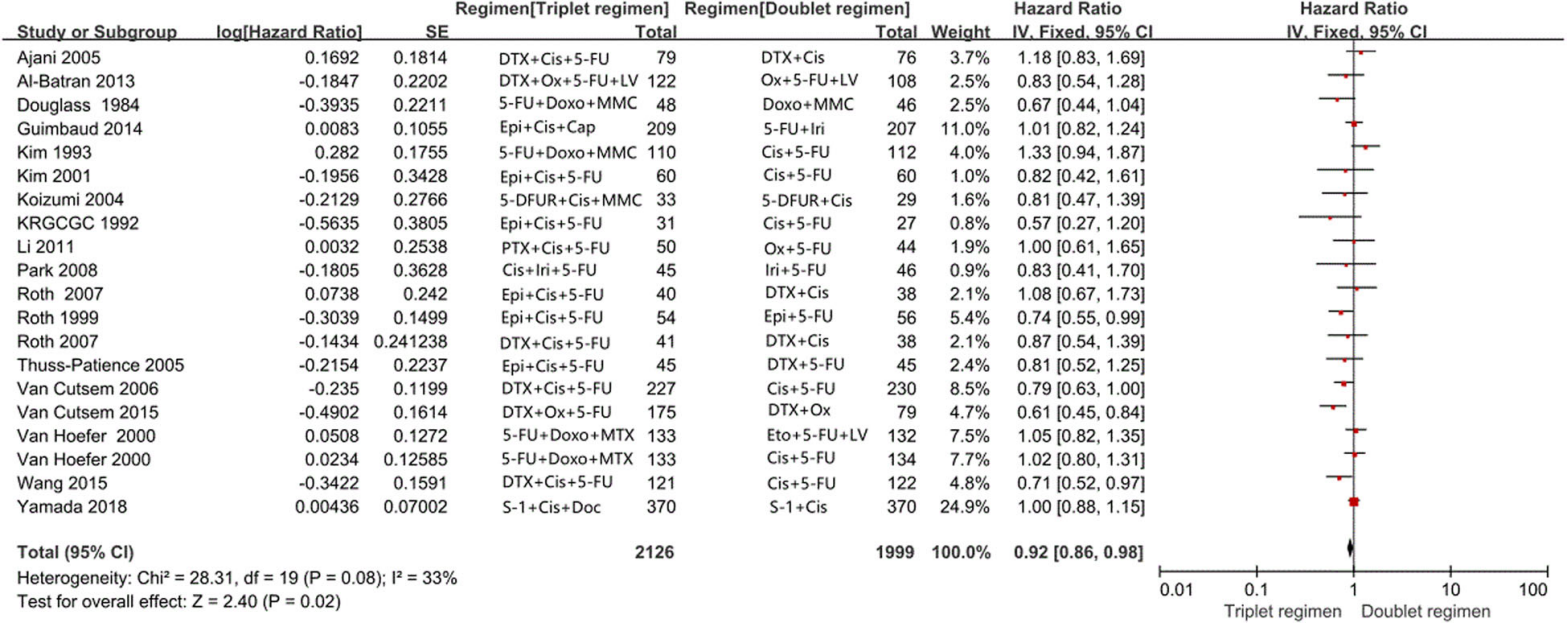
Effects of triplet chemotherapy versus doublet chemotherapy on overall survival

Twenty of the 23 trials reported OS in the study patients. OS was compared in 2126 patients treated wo received triplet chemotherapy with 1999 patients who received doublet chemotherapy. A significant reduction in the risk of death (HR = 0.92; 95% CI, 0.86–0.98; *P* = 0.02) was observed with triplet chemotherapy, as shown in Fig. 2. Heterogeneity in the data was not observed (*P* = 0.08, I^2^ = 33%), which was assessed using a fixed effects model.

**Fig. 3:**
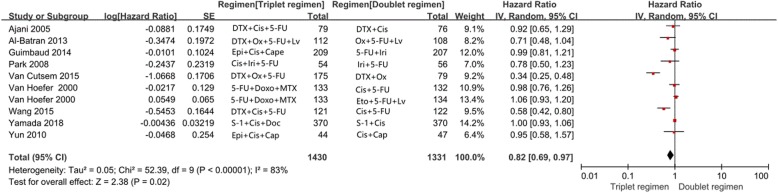
Effects of triplet chemotherapy versus doublet chemotherapy on progression-free survival

Ten of the 23 trials reported PFS in the study patients. The meta-analysis results showed that triplet chemotherapy also significantly improved PFS compared with doublet chemotherapy in patients (HR = 0.82; 95% CI, 0.69–0.97; *P* = 0.02, Fig. 3). Comparison was performed under the random effects model, because obvious heterogeneity was observed (*P* < 0.0001, I^2^ = 83%).

**Fig. 4:**
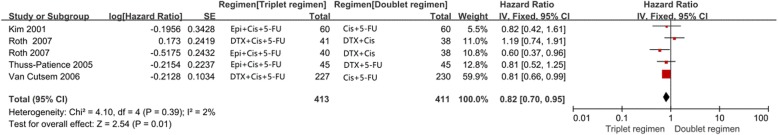
Effects of triplet chemotherapy versus doublet chemotherapy on time to progress

Five out of the 23 trials provided data regarding the TTP, while only one had HR. A meta-analysis was performed using fixed effects model to pool the HRs as there was no heterogeneity among trials (*P* = 0.39, I^2^ = 2%). The combined HR for TTP showed that triplet chemotherapy was superior compared with doublet combination regimen (HR = 0.82; 95% CI, 0.70–0.95; *P* = 0.01, Fig. 4).

**Fig. 5:**
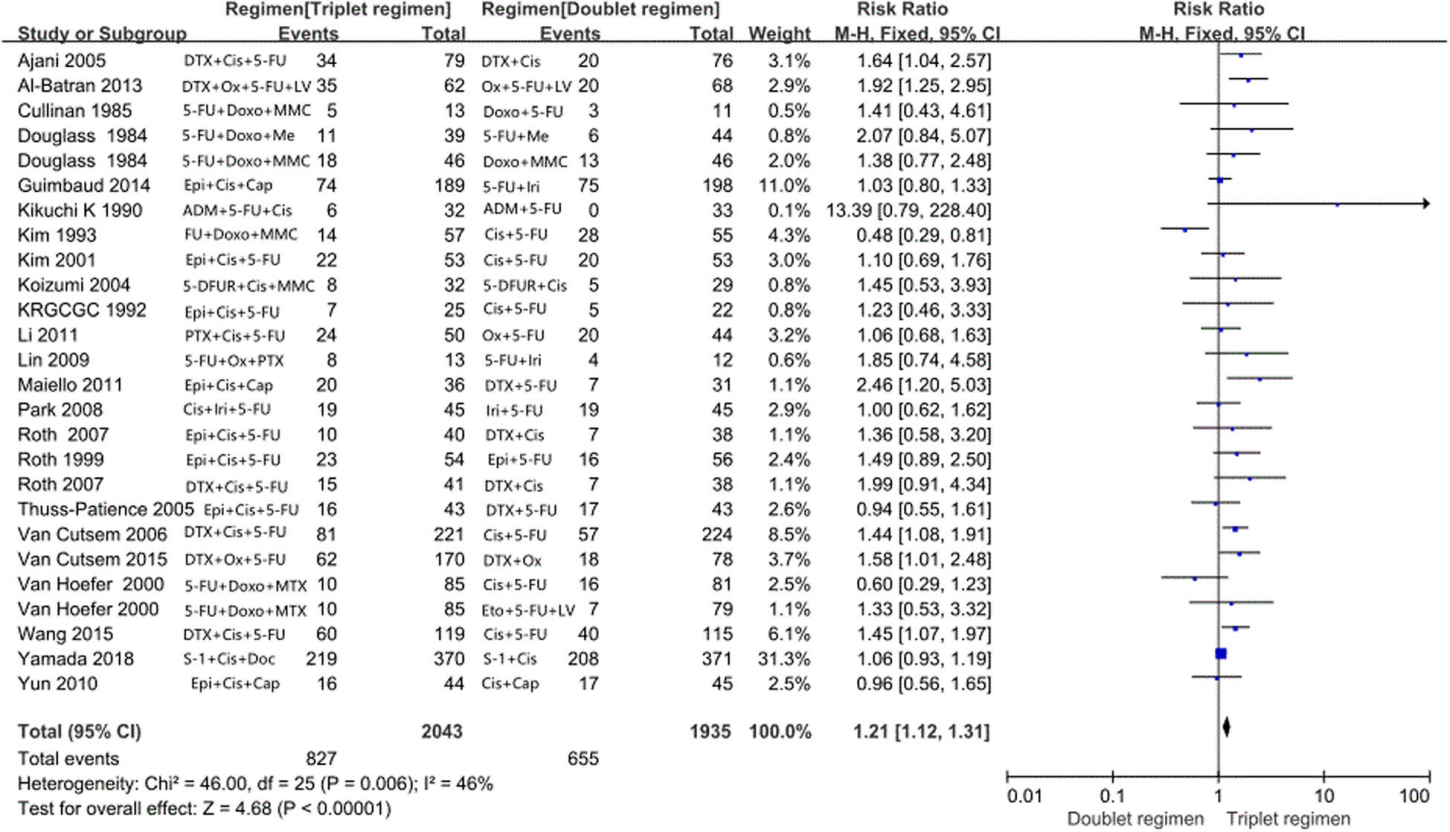
Effect of triplet chemotherapy versus doublet chemotherapy on objective response rate

All the 23 studies demonstrated ORR. The meta-analysis showed a significant improvement for ORR in triplet chemotherapy compared with doublet chemotherapy group (OR = 1.21; 95% CI, 1.12–1.31; *P* < 0.0001, Fig. 5). The I^2^ value of the heterogeneity test was 46%, and a fixed effects model was used.

### Subgroup analysis

We conducted a subgroup analysis according to the comparison of different triplet chemotherapy regimens containing two identical drugs with doublet regimens. Moreover, we also performed a subgroup analysis in patients who were from Asia or the Western. We summarized the results of our subgroup analysis for OS, PFS, and ORR in Additional file 1: Figure S1, Additional file 2: Figure S2 and Additional file 3: Figure S3 (Data not shown).

### Fluoropyrimidine-based triplet versus non-fluoropyrimidine-based doublet chemotherapy

Four trials reported four fluoropyrimidine-based triplet chemotherapy compared with doublet chemotherapy [20, 24, 25, 29]. The results of the subgroup analysis revealed that the addition of fluoropyrimidine in triplet chemotherapy regimens improved OS significantly but not PFS compared with the doublet chemotherapy (HR = 0.80; 95% CI, 0.66–0.96; *P* = 0.02; I^2^ = 63% vs. HR = 0.56; 95% CI, 0.21–1.46; *P* = 0.24; I^2^ = 94%, respectively, Additional file 1: Figure S1, Additional file 2: Figure S2). Additionally, fluoropyrimidine-based triplet regimens had a higher ORR than doublet chemotherapy (OR = 1.60; 95% CI, 1.23–2.09; *P* = 0.0005; I^2^ = 0%, Additional file 3: Figure S3).

### Platinum-based triplet versus non-platinum-based doublet chemotherapy

Among the included trials, three trials reported three platinum-based triplet chemotherapy compared with doublet chemotherapy [23, 36, 40]. The results of the subgroup analysis revealed that the addition of a platinum in triplet chemotherapy regimens had a significant improvement on OS compared with the doublet chemotherapy regimens (HR = 0.75; 95% CI, 0.57–0.99; *P* = 0.04; I^2^ = 0%, Additional file 1: Figure S1). Moreover, platinum-based triplet chemotherapy was not superior in terms of ORR compared with doublet chemotherapy (OR = 1.39; 95% CI, 0.98–1.97; *P* = 0.06; I^2^ = 54%, Additional file 3: Figure S3).

### Anthracycline-based triplet versus non-anthracycline-based doublet chemotherapy

For anthracycline-based regimens, four trials reported the comparison between anthracycline-based triplet chemotherapy and non-anthracycline-based doublet chemotherapy [29, 30, 33, 39]. The results of the subgroup analysis revealed that the addition of an anthracycline in triplet chemotherapy was not associated with a better OS than the doublet chemotherapy (HR = 0.70; 95% CI, 0.42–1.15; *P* = 0.16; I^2^ = 0%, Additional file 1: Figure S1). Anthracycline-based triplet chemotherapy was also not related to better ORR compared with doublet chemotherapy (OR = 1.18; 95% CI, 0.86–1.62; *P* = 0.30; I^2^ = 0%, Additional file 3: Figure S3).

### Mitomycin-based triplet versus non-mitomycin-based doublet chemotherapy

Two trials investigated the treatment difference between MMC-based triplet chemotherapy with non-MMC-based doublet chemotherapy [28, 32]. The results of the subgroup analysis revealed that MMC-based triplet chemotherapy had not an improvement on ORR compared with doublet chemotherapy (OR = 1.43; 95% CI, 0.67–3.08; *P* = 0.36; I^2^ = 0%, Additional file 3: Figure S3).

### Taxane-based triplet versus non-taxane-based doublet chemotherapy

Four trials reported four taxane-based triplet chemotherapy compared with doublet chemotherapy [12, 14, 21, 26, 32]. The results of the subgroup analysis revealed that compared with taxane-based doublet chemotherapy, taxane-based triplet chemotherapy improved neither OS nor PFS (HR = 0.91; 95% CI, 0.81–1.01; *P* = 0.07; I^2^ = 50% vs. HR = 0.76; 95% CI, 0.52–1.11; *P* = 0.16; I^2^ = 85%, respectively, Additional file 1: Figure S1, Additional file 2: Figure S2). However, taxane-based triplet chemotherapy improved significantly the ORR (OR = 1.22; 95% CI, 1.10–1.36; *P* = 0.0002; I^2^ = 75%, Additional file 3: Figure S3).

### Irinotecan-based triplet versus non-irinotecan-based doublet chemotherapy

Considering there was no study comparing irinotecan-based triplet regimens with non-irinotecan-based doublet regimen, there were actually two trials that compared irinotecan-based doublet chemotherapy with irinotecan-based triplet chemotherapy regimens [22, 35], and the subgroup analysis also estimated the different treatment outcomes between the two groups, although the chemotherapeutic drugs in doublet regimens are not identical to triplet regimens. The results of the subgroup analysis revealed that triplet chemotherapy regimens did not improve the ORR (OR = 1.08; 95% CI, 0.85–1.37; *P* = 0.55; I^2^ = 31%, Additional file 3: Figure S3).

### Other triplet versus non-doublet chemotherapies

Eight trials compared other triplet chemotherapies with doublet chemotherapies. Subgroup analysis indicated that triplet chemotherapy did not improve both OS and PFS compared with doublet chemotherapy (HR = 1.05; 95% CI, 0.92–1.21; *P* = 0.46; I^2^ = 0% vs. HR = 1.04; 95% CI, 0.93–1.17; *P* = 0.50; I^2^ = 0%, respectively, Additional file 1: Figure S1, Additional file 2: Figure S2). Moreover, triplet chemotherapy had lower ORR than doublet chemotherapy (HR = 0.95; 95% CI, 0.76–1.19; *P* = 0.66; I^2^ = 63%; Additional file 3: Figure S3).

### Asian and Western patients

**Fig. 6:**
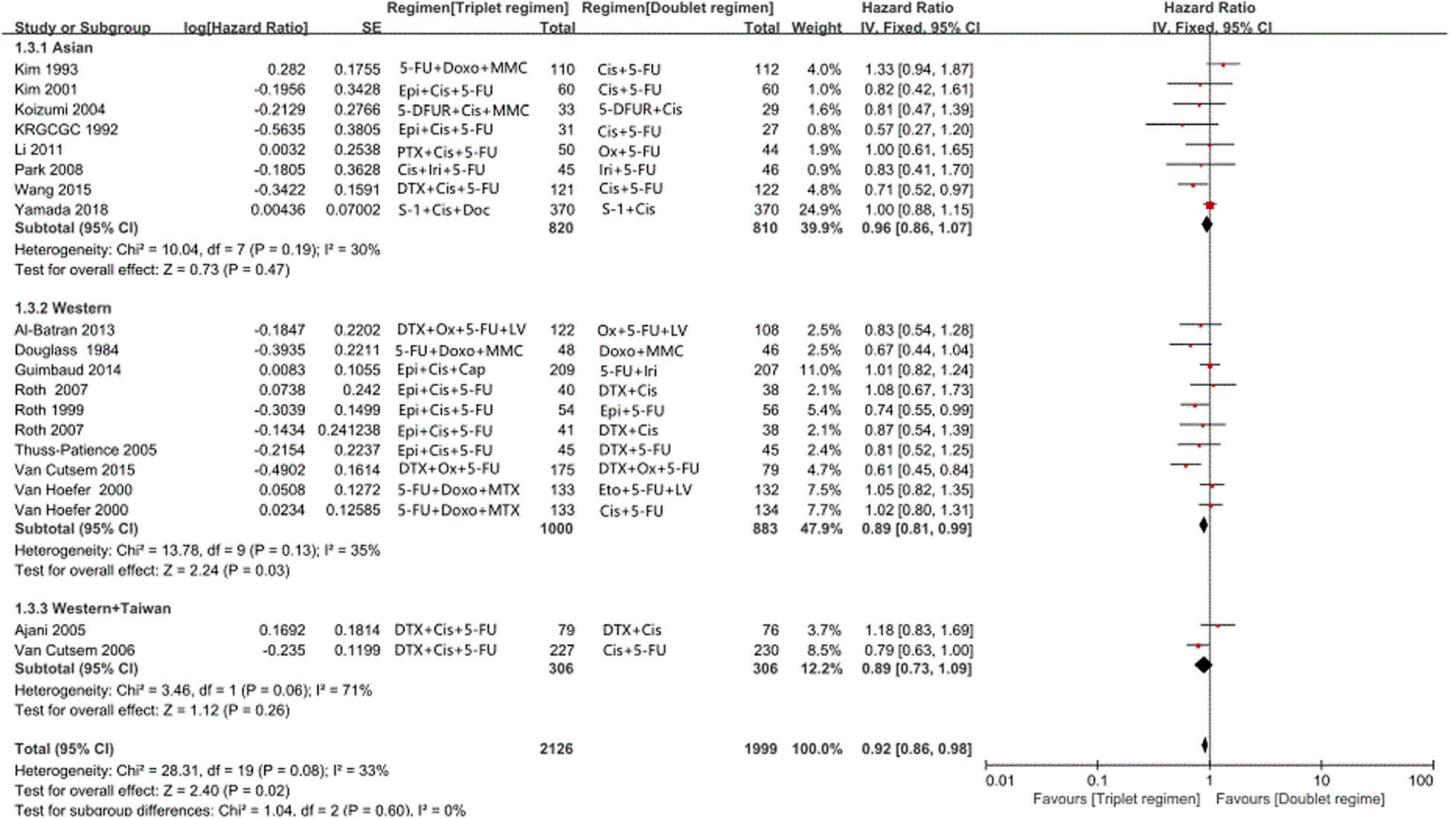
Subgroup analysis of overall survival for triplet regimens compared with doublet regimens between Asian and Western patients

A total of 11 and 10 trials were conducted in Asian and Western patients, respectively. Two other trials were analyzed individually as the included patients were both from Asia and the Western, but detailed geographic data of these patients were not taken. Subgroup meta-analyses based on different patients including Asians and Westerners were further performed (Fig. 6). The results revealed that triplet chemotherapy did not improve OS compared with the doublet chemotherapy (HR = 0.96; 95% CI, 0.86–1.07; *P* = 0.47; I^2^ = 30%) among Asian patients. However, triplet chemotherapy significantly improved OS compared with the doublet chemotherapy (HR = 0.89; 95% CI, 0.81–0.99; *P* = 0.03; I^2^ = 35%) among Western patients.

### Comparison of the same chemotherapy regimens

This meta-analysis included a lot of primary studies compared different doublet and triplet chemotherapy. Considering that the inherent heterogeneity of different chemotherapeutic drugs may affect the results of this meta-analysis, we choose the same chemotherapy regimens between triplet and doublet chemotherapy to carry out subgroup meta-analysis, and studies that have only one type of triplet and doublet chemotherapy regimens were deleted. The results of the subgroup analysis revealed that triplet chemotherapy regimens improve the OS (OR = 0.88; 95% CI, 0.80–0.97; *P* = 0.009; I2 = 48%, Additional file 4: Figure S4) and ORR(OR = 1.26; 95% CI,1.15–1.39; *P* < 0.00001; I2 = 50%, Additional file 6: Figure S6), and PFS has not been further improved (OR = 0.67; 95% CI,0.45–1.00; *P* < 0.00001; I2 = 92%, Additional file 5: Figure S5).

### Publication bias

**Fig. 7:**
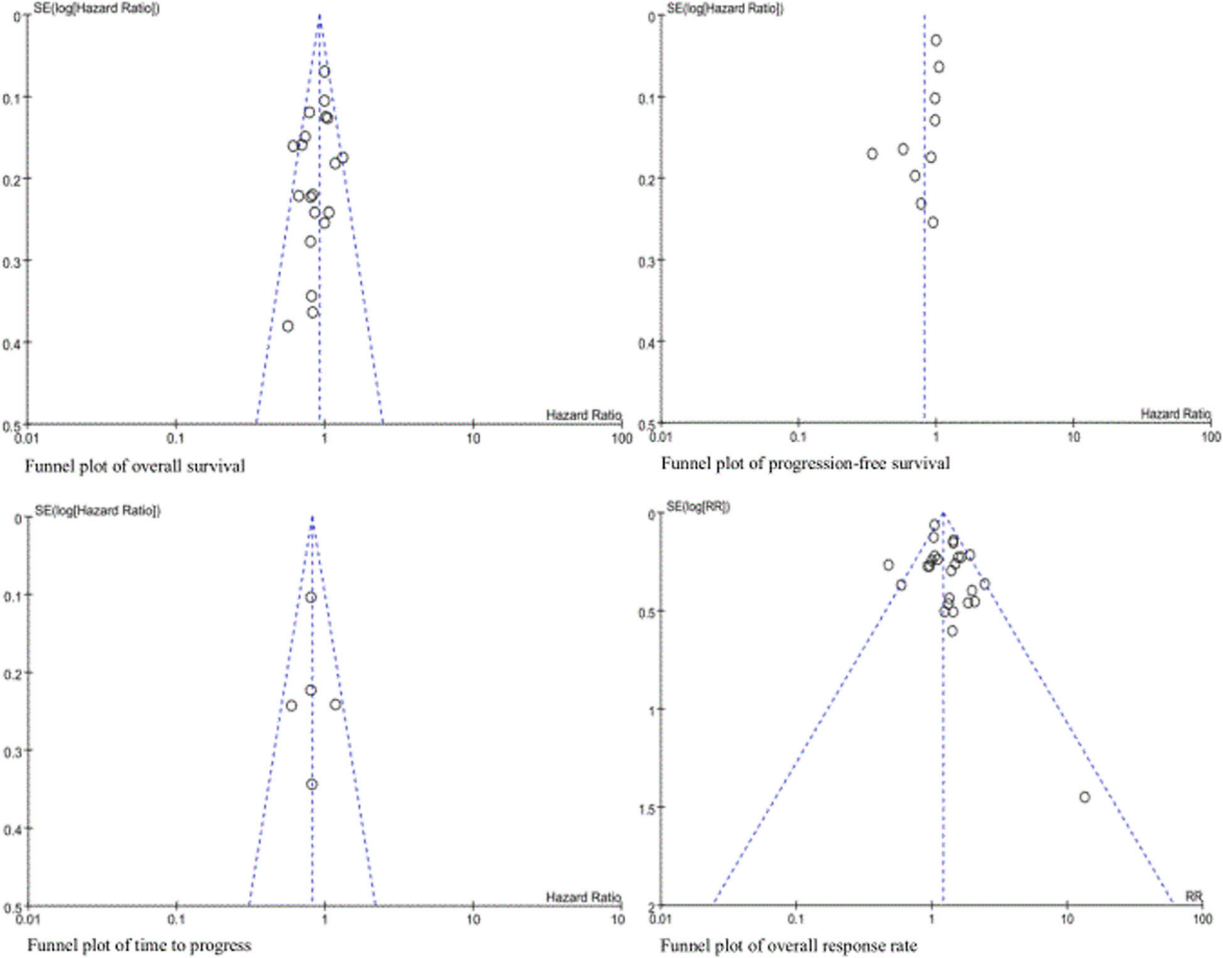
Risk of bias assessment

The funnel plots did not show significant asymmetry for triplet versus doublet chemotherapy in terms of OS, PFS, TTP, and ORR (Fig. 7).

### Toxicities

Main data were available for 5 hematological, 16 nonhematological, and 4 laboratory-assessed items among the 23 trials. We summarized grade 1–2 and grade 3–4 AEs, and the results are shown in Table 2. The most common grade 3–4 hematological toxicities were neutropenia and leucopenia, while the most common nonhematological toxicities were nausea, vomiting, diarrhea, stomatitis, anorexia, fatigue, alopecia, and lethargy. There were significantly more incidences of grade 3–4 neutropenia (RR = 1.46; 95% CI, 1.32–1.60; *P* < 0.001), leucopenia (RR = 1.51; 95% CI, 1.33–1.71; *P* < 0.001), febrile neutropenia (RR = 1.87; 95% CI, 1.33–2.62; *P* < 0.001), diarrhea (RR = 1.68; 95% CI, 1.25–2.25; *P* < 0.001), and infection (RR = 1.80; 95% CI, 1.11–2.92; *P* = 0.02) in triplet chemotherapy group compared with combination chemotherapy group, while equivalent frequencies of grade 3–4 AEs were found between the two groups.

**Table 2:** Toxicity results of triplet chemotherapy compared with doublet chemotherapy

Toxicity Category	Grade 1 or 2								Grade 3 or 4							
	Triplet			Doublet					Triplet			Doublet				
	T	Total	%	T	Total	%	RR	95%CI	T	Total	%	T	Total	%	RR	95%CI
Hematological																
Neutropenia	131	748	18	184	651	28	0.62	0.51-0.76	682	1234	55	368	970	38	1.46	1.32-1.60
Leucopenia	291	680	42	277	684	40	1.06	0.93-1.20	474	1102	43	252	885	28	1.51	1.33-1.71
Anemia	498	925	53	454	924	49	1.10	1.00-1.20	111	784	14	123	682	18	0.79	0.62-0.99
Thrombocytopenia	151	846	18	162	848	19	0.93	0.76-1.14	113	1181	9	119	934	12	0.75	0.59-0.96
Febrile neutropenia	79	254	31	46	214	21	1.45	1.06-1.98	109	1026	10	44	774	6	1.87	1.33-2.62
Non-hematological																
Nausea	358	584	61	353	491	71	0.85	0.78-0.93	115	1130	10	118	900	13	0.78	0.61-0.99
Vomiting	295	716	41	275	622	44	0.93	0.82-1.06	74	795	9	70	618	11	0.82	0.60-1.12
Diarrhea	343	748	46	201	651	31	1.49	1.29-1.71	125	1590	8	62	1323	5	1.68	1.25-2.25
Stomatitis	254	716	35	165	622	27	1.34	1.14-1.58	151	1594	9	96	923	10	0.91	0.71-1.16
Anorexia	150	467	32	138	376	36	0.88	0.73-1.06	57	546	10	41	452	9	1.15	0.79-1.69
Fatigue	175	376	46	132	283	47	1.00	0.85-1.18	52	376	14	35	283	12	1.12	0.75-1.67
Hand foot yndrome	45	259	17	32	168	19	0.91	0.61-1.37	17	376	5	7	237	3	1.53	0.64-3.64
Sensory europathy	227	597	38	162	507	32	1.19	1.01-1.40	75	1021	7	49	805	6	1.21	0.85-1.71
Alopecia	83	242	34	39	148	26	1.30	0.94-1.97	87	538	16	45	274	16	0.98	0.71-1.37
Pigmentation	33	215	15	11	123	9	1.72	0.90-3.27	8	296	3	7	161	4	0.62	0.23-1.68
Lethargy	83	221	37	74	224	33	1.14	0.88-1.46	57	300	19	45	300	15	1.27	0.89-1.81
Infected	21	293	7	22	294	7	0.96	0.54-1.70	41	291	14	23	294	78	1.80	1.11-2.92
Constipation	62	286	22	41	193	21	1.02	0.72-1.45	0	286	0	5	193	0.06	0.06	0.00-1.10
Fluid retention	17	72	24	10	70	14	1.65	0.81-3.36	1	72	1.3	3	70	4	0.32	0.03-3.04
Allergy	5	72	7	5	70	7	0.97	0.29-3.21	1	72	1.3	1	70	1.3	0.97	0.06-15.24
Abdominal pain	33	170	19	21	78	27	0.72	0.45-1.16	6	170	3.5	4	78	5.1	0.69	0.20-2.37
Laboratory-assessed items																
Increased ALT	28	149	19	28	144	19	0.97	0.60-1.55	0	149	0	1	144	0.6	0.32	0.01-7.85
Increased AST	35	104	34	21	99	21	1.59	1.00-2.53	0	104	0	1	99	1	0.32	0.01-7.70
Increased ALP	24	72	33	27	70	38	0.86	0.56-1.34	1	72	1.3	2	70	2.8	0.49	0.05-1.40
Creatinine	18	149	12	26	144	18	0.67	0.38-1.17	1	149	0.6	0	144	0	2.90	0.12-7.61

*ALT* Alanine aminotransferase, *AST* Aspartate aminotransferase, *ALP* alkaline phosphatase

## Discussion

The debate of triplet or doublet chemotherapy in treating patients with advanced gastric cancer has been existing for a long time, which started from the 1980s. Most of the earliest studies of triplet and doublet chemotherapy contained drugs, such as FU, Doxo, MMC, and Eto. With the development of the novel chemotherapeutic drugs, triplet and doublet chemotherapy regimens contained additional new drugs such as Epi, Iri, Taxa, Cap, Ox, and T in triplet or doublet chemotherapy in treating advanced gastric cancer.

Though nearly 30 RCTs were conducted, whether triplet or doublet chemotherapy improves the survival of patients with advanced gastric cancer remains unclear. The results were also identical among meta-analyses [8, 9, 41]. TTP in all patients with advanced gastric cancer. The result of OS and PFS was in line with the previous meta-analyses [9]. We enrolled all RCTs from the 1980s to October, 2018 and strictly and separately finished pooled analysis of PFS and TTP among 23 trials. A previous meta-analysis emulates PFS and TTP together [9]. Considering the difference of definition and clinical significance, pooled TTP analysis was individually made among included trials. Triplet regimens were in favor of longer TTP compared with doublet chemotherapy. Additionally, as expected, triplet regimens could result to a higher ORR than doublet regimens.

Fluorouracil-based, platinum-based, MMC-based, and anthracycline-based chemotherapies were the early regimens in treating patients with treatment-naive advanced gastric cancer in RCTs [28, 29]. The common doublet regimens include Cis plus FU, Doxo plus FU, FU plus Me, and Epi plus FU. A third drug that was added in the triplet regimens was usually Doxo, FU, Me, Eto, or MMC. The median OS in doublet regimen groups ranged from 3.3 months to 8.61 months, while that in triplet groups was between 5.5 months and 8.5 months [29, 30]. The ORR in doublet regimen groups ranged from 0 to 51% [30, 40], while that in triplet groups ranged from 12 to 39% [27, 29]. A serious new generation of chemotherapeutic drugs such as Epi, DTX and PTX, Ox, Iri, Cap, and S-1 were also added into doublet or triplet chemotherapy in treating patients with advanced gastric cancer. Epi, Cap or S-1, and Ox replace Doxo, FU, and Cis in new doublet regimens, respectively. Also, DTX or PTX and Iri were added in novel doublet regimens, respectively. Similarly, a third new chemotherapeutic drug was added into traditional or new doublet regimens, resulting in a series of new triplet chemotherapy regimens. These new triplet regimens were widely compared with traditional or new doublet regimens in various RCTs in advanced gastric cancer. The common triplet regimens include Epi plus Cis plus 5-FU/Cap, DTX/PTX plus Cis/Ox plus 5-FU/Cap/S-1, and Cis plus Iri plus FU. The new doublet regimens have an OS that ranged from 7.1 to 15.3 months and an ORR that ranged from 18.4 to 56% [12, 24, 36]. The triplet regimens have an OS that ranged from 8.3 to 17.3 months and an ORR that ranged from 27 to 59.3% [12, 21, 24, 33]. Both OS and ORR were significantly improved in new doublet and triplet regimens [2, 42].

There were more than 20 triplet regimens and doublet regimens that were included in this meta-analysis. We divided these chemotherapy regimens into seven kinds, that is, whether two of the chemotherapeutic drugs present in triplet regimens were identical or homogenous to doublet regimens. These regimens included fluorouracil-based, platinum-based, MMC-based, anthracycline-based, taxane-based, and other chemotherapies. Because of the absence of a study that compares irinotecan-based triplet regimens with non-irinotecan-based doublet regimen, we also classified a kind of “irinotecan,” that is, irinotecan-based double regimens.

This systematic review and meta-analysis revealed that fluorouracil-based triplet regimens were superior to doublet regimens in terms of OS and ORR but not PFS. These results were consistent with the previous meta-analysis [9]. The pooled result of the improved PFS in fluorouracil-based triplet chemotherapy was not completely convincing due to the following reasons: high heterogeneity and relatively small samples. The HR (0.80) of OS may still be probable and is considered clinically meaningful because of the presence of relatively large samples. Platinum-based triplet regimens improved OS but not PFS and ORR compared with doublet regimen. These results were in line with previous meta-analysis and also were similar with another. However, MMC-based and anthracycline-based triplet regimens improved neither primary nor second outcomes. What should be noticed is that the results of the pooled analysis of anthracycline-based triplet regimens benefiting patients with advanced gastric cancer remain controversial. An early meta-analysis confirmed that anthracycline-based triplet regimens could improve OS [41]. Nevertheless, a recent meta-analysis holds the doubtful conclusion [9]. Moreover, another recent network meta-analysis indicates that anthracycline-based triplet chemotherapy did not improve OS and PFS compared with fluorouracil-based doublet chemotherapy [8]. Though our meta-analysis included RCTs and had no heterogeneity, the overall patient samples were small. Thus, it is still hard to confirm if patients did benefit from anthracycline-based triplet regimens.

In our meta-analysis, taxane-based triplet regimens did not improve OS but improved ORR for patients with advanced gastric cancer. Whether taxane-based triplet regimens improve survival is the mostly disputed topic among previous meta-analysis. A meta-analysis concluded that taxane-based triplet regimens significantly improved OS, PFS, and ORR of patients with advanced gastric cancer [9]. However, a network meta-analysis revealed that taxane-based triplet regimens did not improve OS and PFS compared with fluorouracil-based doublet chemotherapy [8]. The former included one more trial than the latter. Additionally, our meta-analysis also enrolled new large samples of an RCT accounting for 24.9% of all included trials [12]. The different RCT samples among several meta-analysis contributed the various outcomes. A more recent study with 741 patients failed to prove that taxane-based triplet regimens could improve OS, PFS, and ORR compared with doublet regimens [12]. This study had majority of weight of taxane-based subgroup in our meta-analysis and was related to the negative outcome of OS. Nevertheless, our pooled analysis still demonstrated that taxane-based triplet regimens improved ORR of patients with advanced gastric cancer. Lastly, other drug-based regimens did not improve OS, PFS, and ORR in patients with advanced gastric cancer, and irinotecan-based chemotherapy regimens also did not improve the ORR.

To the best of our knowledge, this meta-analysis firstly and separately analyzed Asian and Western patients, that is, whether they can get more benefit from triplet chemotherapy compared with doublet chemotherapy. The pooled result revealed that Western patients’ OS improved with triplet chemotherapy while Asian patients’ OS did not. There were 11 trials including 1630 patients and 10 trials including 1883 patients in Asia and Western, respectively, in our meta-analysis. Moreover, both subgroups had low heterogeneity (I^2^ = 30% in Asia and 35% in Western group). We also individually analyze two trials as a subgroup that included patients both from Asia and Western; however, detailed geographic data of patients were not taken. Therefore, the results of the different improvement of OS between Asian and Western patients could be highly robust. Studies have shown that the proportion of patients with advanced gastric cancer in Asia receiving second-line treatment were higher than that in Western patients [43–48]. Furthermore, a meta-analysis showed that the 1-year OS rate of advanced gastric cancer will improve by 10% for every 10% increase in patients receiving second-line chemotherapy [49]. And the first-line use of triplet chemotherapy may lead to drug resistance to basic chemotherapeutic drugs and reduce the choice of follow-up chemotherapeutic drugs. Hence, it is most likely that further treatment following the first-line treatment in Asia confounded the outcomes of triplet combination chemotherapy.

Subgroup analysis of the same chemotherapy regimens indicated that triplet chemotherapy regimens improve the OS and ORR, while PFS had negative result. The overall PFS analysis showed that triplet chemotherapy regimens could significantly improve PFS, but the subgroup analysis of the same regimens showed the negative result, which may be related to deletion of studies that have only one type of triplet and doublet chemotherapy regimens.

Some limitations of the present analysis should be acknowledged. First is the difference in the parameters of patients, regimens, and dose induced to heterogeneity among some of the included trials. Though we used the random effects model to compute the estimates, the heterogeneity might potentially affect the results. Second, patients receiving second-line treatments were not reported; hence, the possible impact on outcomes could not be considered. However, second-line treatments were not related to the PFS in first-line chemotherapy. Third, our meta-analysis was based on the aggregate data from longitudinal RCTs rather than individual patient data. Therefore, discrimination in individual baseline parameters cannot be regulated. Fourth, some of the included trials in our analysis did not provide the data of OS, PFS, TTF, and toxicity, especially several abstracts from ASCO and ESMO conferences. Insufficient amount of data might potentially influence the analysis.

## Conclusion

In conclusion, compared with doublet chemotherapy, triplet chemotherapy, as a first-line treatment, improved OS, PFS, TTP, and OS in patients with advanced gastric cancer among overall populations, especially for fluoropyrimidine- or platinum-based triplet chemotherapy, which showed a significant improvement in OS. In the subgroup analyses, triplet chemotherapy improved OS in Western but not in Asian patients.

## Supplementary information

**Additional file 1: Figure S1.** Subgroup analysis of overall survival for triplet chemotherapy versus doublet chemotherapy.

**Additional file 2: Figure S2.** Subgroup analysis of progression-free survival for triplet chemotherapy versus doublet chemotherapy.

**Additional file 3: Figure S3.** Subgroup analysis of objective response rate for triplet chemotherapy versus doublet chemotherapy.

**Additional file 4: Figure S4.** Comparison of the same chemotherapy regimens of overall survival for triplet chemotherapy versus doublet chemotherapy.

**Additional file 5: Figure S5.** Comparison of the same chemotherapy regimens of progression-free survival for triplet chemotherapy versus doublet chemotherapy.

**Additional file 6: Figure S6.** Comparison of the same chemotherapy regimens of objective response rate for triplet chemotherapy versus doublet chemotherapy.

## Data Availability

Not applicable.

## Abbreviations

5-FU: 5-fluorouracil
AEs: Adverse events
ASCO: American Society of Clinical Oncology
Cap: Capecitabine
CENTRAL: Cochrane Register of Controlled Trials
Cis: Cisplatin
D: Doxorubicin
DCF: Docetaxel and cisplatin plus fluorouracil
E: Epirubicin
E: Etoposide
ECOG: Eastern Cooperative Oncology Group
ESMO: European Society for Medical Oncology
F: Fluoropyrimidine
HER2: Human epidermal growth factor receptor 2
I: Irinotecan
ITT: Intention-to-treat
Me: Semustine
MMC: Mitomycin
Mtx: Methotrexate
ORR: Objective response rate
Ox: Oxaliplatin
PFS: Progression-free survival
PRISMA: Preferred Reporting Items for Systematic Reviews and Meta-Analyses
PS: Performance status
RCTs: Randomized controlled trials
SE: Standard error
T: Taxane
Te: Tegafur
TTP: Time to progress
U: Uracil
