# Supplementary figures and images for: A comparison between triplet and doublet chemotherapy in improving the survival of patients with advanced gastric cancer: a systematic review and meta-analysis
Source: BMC Cancer. 2019 Nov 20;19:1125. doi: 10.1186/s12885-019-6294-9 (PMC6865072; doi:10.1186/s12885-019-6294-9)

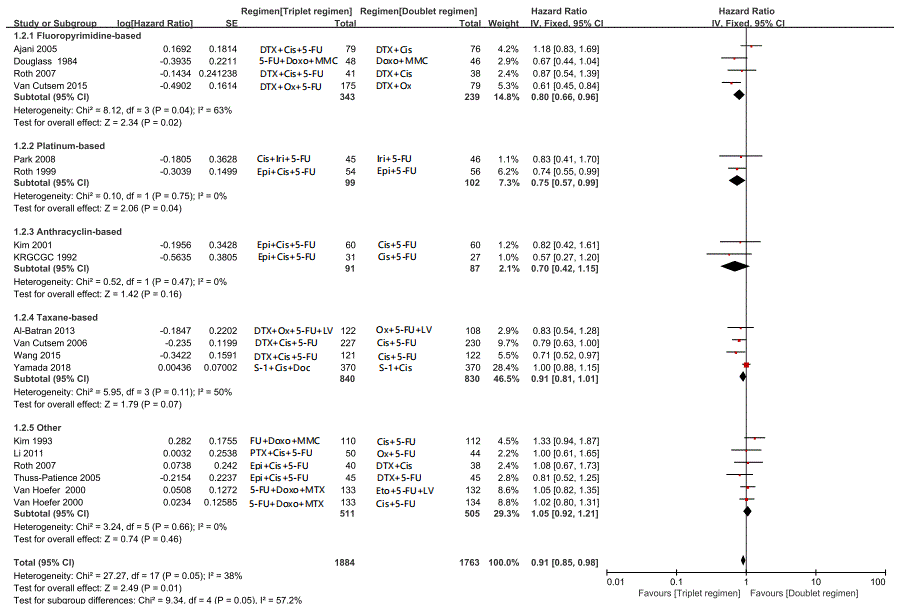

Supplement: Supplementary file 1 — Additional file 1: Figure S1. Subgroup analysis of overall survival for triplet chemotherapy versus doublet chemotherapy. [file 12885_2019_6294_MOESM1_ESM.tif]

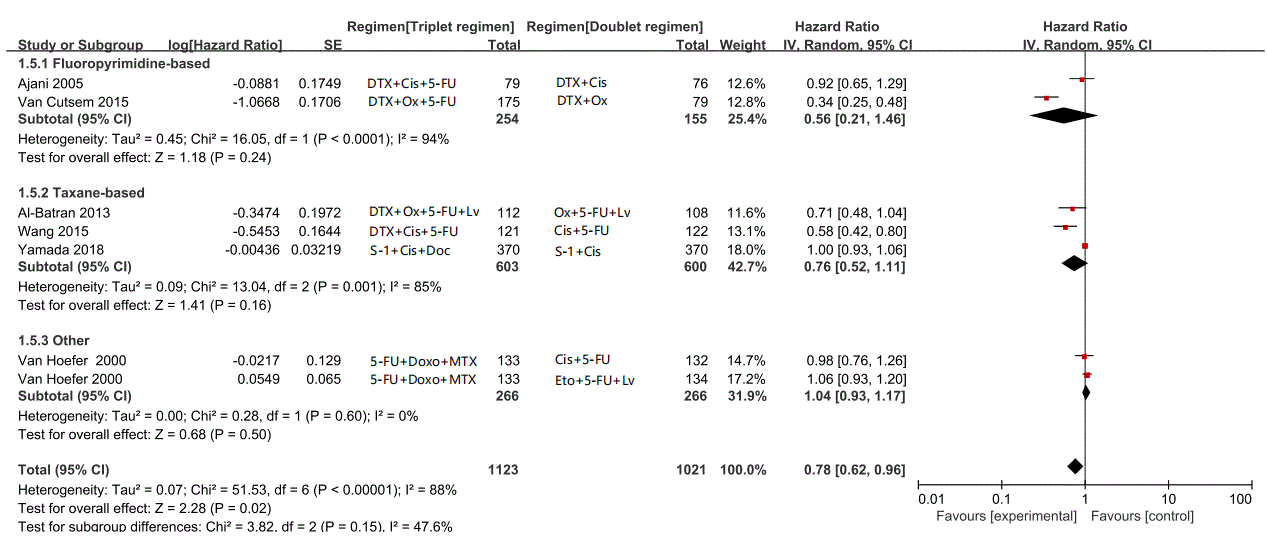

Supplement: Supplementary file 2 — Additional file 2: Figure S2. Subgroup analysis of progression-free survival for triplet chemotherapy versus doublet chemotherapy. [file 12885_2019_6294_MOESM2_ESM.tif]

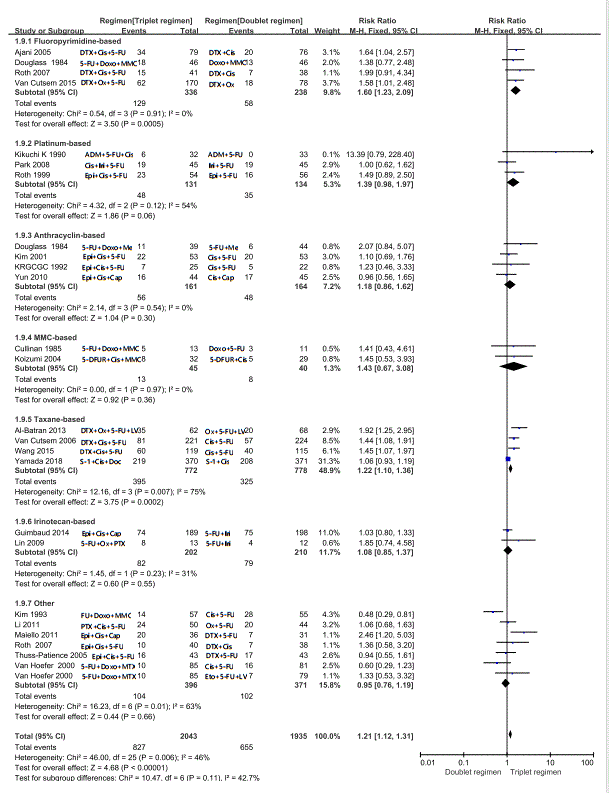

Supplement: Supplementary file 3 — Additional file 3: Figure S3. Subgroup analysis of objective response rate for triplet chemotherapy versus doublet chemotherapy. [file 12885_2019_6294_MOESM3_ESM.tif]

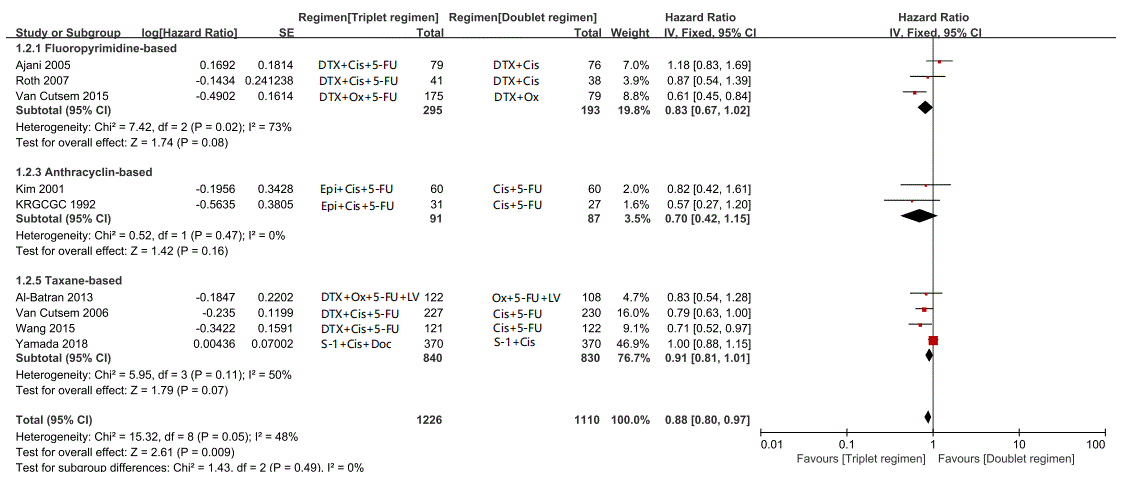

Supplement: Supplementary file 4 — Additional file 4: Figure S4. Comparison of the same chemotherapy regimens of overall survival for triplet chemotherapy versus doublet chemotherapy. [file 12885_2019_6294_MOESM4_ESM.tif]

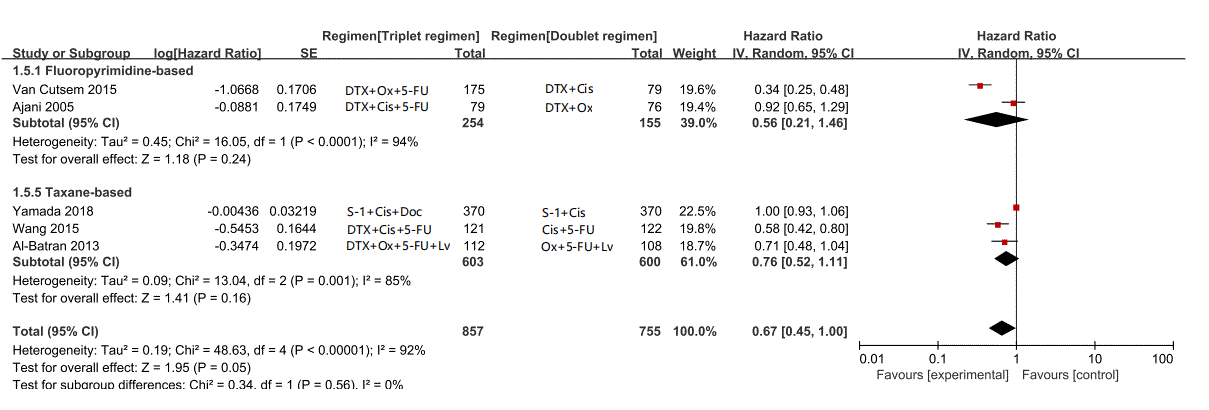

Supplement: Supplementary file 5 — Additional file 5: Figure S5. Comparison of the same chemotherapy regimens of progression-free survival for triplet chemotherapy versus doublet chemotherapy. [file 12885_2019_6294_MOESM5_ESM.tif]

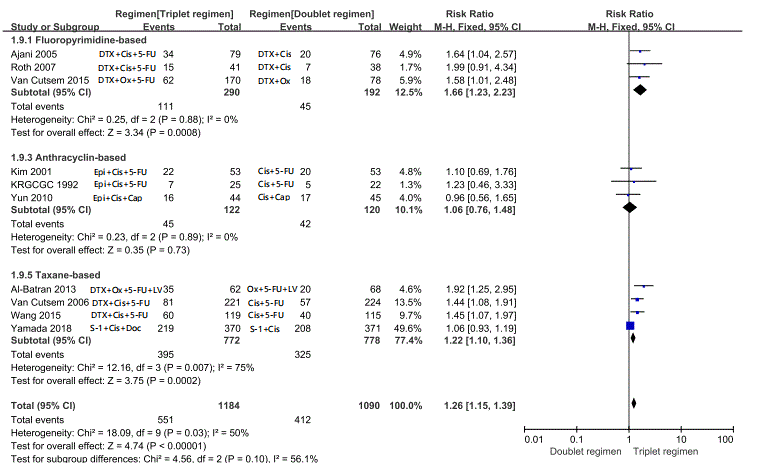

Supplement: Supplementary file 6 — Additional file 6: Figure S6. Comparison of the same chemotherapy regimens of objective response rate for triplet chemotherapy versus doublet chemotherapy. [file 12885_2019_6294_MOESM6_ESM.tif]
